# Vaccination against Extracellular Vimentin for Treatment of Urothelial Cancer of the Bladder in Client-Owned Dogs

**DOI:** 10.3390/cancers15153958

**Published:** 2023-08-03

**Authors:** Diederik J. M. Engbersen, Judy R. van Beijnum, Arno Roos, Marit van Beelen, Jan David de Haan, Guy C. M. Grinwis, Jack A. Schalken, J. Alfred Witjes, Arjan W. Griffioen, Elisabeth J. M. Huijbers

**Affiliations:** 1 CimCure BV, 1081 HV Amsterdam, The Netherlands; djm.engbersen@cimcure.com (D.J.M.E.); j.vanbeijnum@amsterdamumc.nl (J.R.v.B.); ejm.huijbers@cimcure.com (E.J.M.H.); 2 Angiogenesis Laboratory, Department of Medical Oncology, Cancer Center Amsterdam, Amsterdam UMC, 1081 HV Amsterdam, The Netherlands; j.d.dehaan@amsterdamumc.nl; 3 Veterinary Referral Center Korte Akkeren, 2802 LA Gouda, The Netherlands; arno.roos@evidensia.nl (A.R.); marit.van.beelen@evidensia.nl (M.v.B.); 4 Veterinary Pathology Diagnostic Centre, Department of Biomedical Health Sciences, Faculty of Veterinary Medicine, Utrecht University, 3584 TD Utrecht, The Netherlands; g.c.m.grinwis@uu.nl; 5 Department of Urology, Radboud University Medical Center, 6525 GA Nijmegen, The Netherlands; jack.schalken@radboudumc.nl (J.A.S.); fred.witjes@radboudumc.nl (J.A.W.)

**Keywords:** angiogenesis, antiangiogenic therapy, bladder cancer, immunotherapy, vimentin, tumor vascular marker, vaccination

## Abstract

**Simple Summary:**

In this study, we investigated the safety and usefulness of treatment of dog patients with spontaneous bladder cancer with the CVx1 vaccine. The vaccine is directed against a specific protein, named extracellular vimentin, excreted by the tumor vasculature. Twenty dogs diagnosed at the veterinary clinic with bladder cancer were treated with the CVx1 vaccine in combination with the non-steroidal anti-inflammatory drug (NSAID) meloxicam. All dogs responded to the treatment and developed an immune response towards the tumor vasculature. After treatment with the CVx1 vaccine plus meloxicam, the survival was almost doubled (374 days) compared to the historical control group (196 days) treated with the chemotherapy carboplatin in combination with the NSAID piroxicam. Treatment with the CVx1 vaccine combined with meloxicam was safe and well tolerated. Our results justify further development of the CVx1 vaccine for the treatment of human patients in the clinic.

**Abstract:**

It was recently shown that targeting extracellular vimentin (eVim) is safe and effective in preclinical models. Here, we report the safety and efficacy in client-owned dogs with spontaneous bladder cancer of CVx1, an iBoost technology-based vaccine targeting eVim in combination with COX-2 inhibition. This was a single-arm prospective phase 1/2 study with CVx1 in 20 client-owned dogs with spontaneous UC which involved four subcutaneous vaccinations with CVx1 at 2-week intervals for induction of antibody titers, followed by maintenance vaccinations at 2-month intervals. Additionally, daily cyclooxygenase (COX)-2 inhibition with meloxicam was given. The response was assessed by antibody titers, physical condition, abdominal ultrasound and thorax X-ray. The primary endpoints were the development of antibody titers, as well as overall survival compared to a historical control group receiving carboplatin and COX-2 inhibition with piroxicam. Kaplan–Meier survival analysis was performed. All dogs developed antibodies against eVim. Titers were adequately maintained for the duration of this study. A median overall survival of 374 days was observed, which was 196 days for the historical control group (*p* < 0.01). Short-term grade 1–2 toxicity at the injection site and some related systemic symptoms peri-vaccination were observed. No toxicity was observed related to the induced antibody response. A limitation of this study is the single-arm prospective setting. CVx1 plus meloxicam consistently induced efficient antibody titers, was well tolerated and showed prolonged survival. The results obtained merit further development for human clinical care.

## 1. Introduction

Bladder cancer is the 10th most common cause of cancer affecting 1 in 100 men and 1 in 400 women in life yet carrying the highest lifetime cost of all cancers [1,2,3,4,5]. In addition, current therapy options leave room for improvement [6]. To date, the treatment of muscle-invasive bladder cancer (MIBC) shows an average 5-year survival of 50%, except for patients with metastatic disease, who have a dismal 5-year survival of 5% [7,8]. Recent advances with the use of immune checkpoint inhibitors, namely programmed death 1 (PD-1)/programmed death ligand 1 (PD-L1) inhibitors, have resulted in encouraging, albeit limited, clinical benefit [9,10,11]. Also, many of the tumor vaccine modalities developed for human use have been studied in dogs, and the first tumor vaccine licensed for veterinary use is a DNA-plasmid technology-based vaccine for the treatment of canine malignant melanoma [12,13].

Recently, extracellular vimentin (eVim) has been described as a target for antiangiogenic immunotherapy [14]. eVim is excreted by the tumor endothelial cells of most, if not all, solid tumors and promotes angiogenesis as well as immune suppression. Passive targeting of eVim by monoclonal antibody therapy specifically and safely inhibited tumor growth in mouse melanoma and colorectal tumor models [14,15]. In order to induce endogenous antibody responses to self-antigens, we developed a novel conjugate vaccine technology, named iBoost [16,17] (Figure S1). Using iBoost vaccinations against eVim in mouse melanoma and colorectal carcinoma models, we observed potent anti-tumor responses characterized by reduced tumor growth, diminished tumor blood vessel density and a more favorable intratumoral immune cell composition [14]. Importantly, no side effects as well as no impairment of skin wound healing were observed. Furthermore, in mice that were kept hyperimmune for over one year, body weight, behavior and organ morphology were normal [14]. Here, we present the data of a safety and efficacy study of the anti-eVim CVx1 vaccine in client-owned dogs with spontaneous urothelial carcinoma (UC). In >90% of the cases, dogs presenting with UC have intermediate to high-grade invasive urothelial carcinoma at diagnosis [18]. Bladder cancer in dogs is recognized as a unique and well-documented cancer type that highly resembles human bladder cancer [19,20]. The aim of this study was to evaluate the safety, antibody response and efficacy of the anti-eVim CVx1 vaccine combined with standard COX-2 inhibition in client-owned dogs with spontaneous bladder cancer and to compare the overall survival to the results reported in the literature for treatment with carboplatin plus COX-2 inhibition [21]. We show that CVx1 is well tolerated, induces efficient antibody responses, shows a 100% clinical benefit rate and significantly doubles the median overall survival compared to the historical control group.

## 2. Materials and Methods

### 2.1. Study Design and Participants

#### Study Design

**Figure 1:**
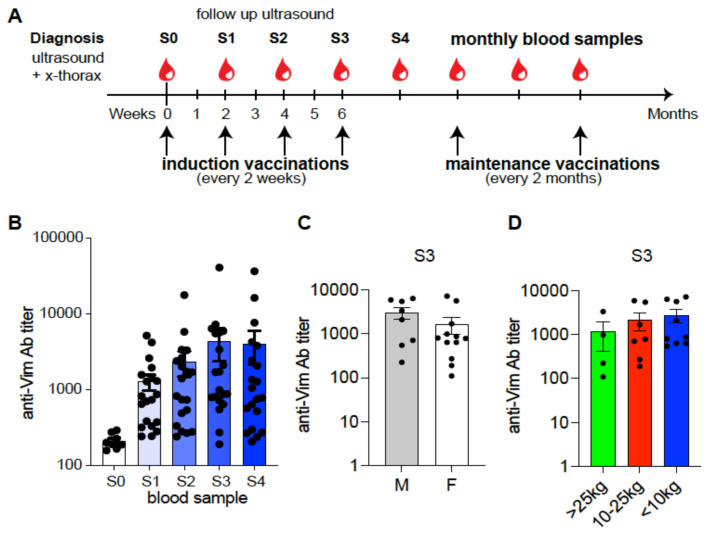
Antibody response in dogs vaccinated with CVx1. (**A**) Schematic overview of the study set-up. An ultrasound of the bladder and X-ray of the thorax were performed at diagnosis. Induction vaccinations were given in 2-week intervals with maintenance vaccinations every 2 months. During the induction phase, blood samples were taken every two weeks (S0–S3), at the time point of vaccination and during maintenance monthly blood samples (S4–S∞) were taken. (**B**) Anti-eVim antibody titers at start of treatment (S0) and after each induction vaccination (S1–S4). (**C**) Anti-eVim antibody titers divided by gender (male (M) *n* = 8; female (F) *n* = 12) at time point S3. (**D**) Anti-eVim antibody titers divided by body weight (>25 kg (green bar) *n* = 4; 10–25 kg (red bar) *n* = 7; <10 kg (blue bar) *n* = 9). Data are depicted as mean ± SEM.

An open-label single-arm prospective clinical study in privately owned dogs was performed following approval by the Animal Ethics Committee of the VU University and the national Central Animal Experiments Committee (reg. no. CCDAVD11400202011305). The ethical approval was granted based on the performance of exclusively routine treatment. This study was designed to assess the safety and efficacy of CVx1 immunotherapy and meloxicam in dogs with urothelial carcinoma in comparison to a historical control group treated with carboplatin and piroxicam [21]. Dogs with histologically/cytologically confirmed primary or recurrent UC with or without metastasis and adequate renal (creatinine > 212 μM/L) and liver (ALT < 125 U/L) functions were eligible. Dogs with recent (two weeks prior to first vaccination) or current treatment with immune suppressive therapy for other diseases and/or prior malignancies were excluded per the principal investigator’s discretion. Upon owner consent, dogs were included in this study; these dogs were not hospitalized and lived at home with their owners. After diagnosis, an ultrasound of the abdomen including the bladder and an X-ray of the thorax were performed at the start of this study and during follow-up. After the initial vaccination, the dogs received 3 booster vaccinations at 2-week intervals followed by maintenance vaccinations at 2-month intervals for the duration of this study and beyond (Figure 1A). Two-week vaccination intervals were chosen because this was important to make the dogs hyperimmune at a fast pace, and shorter intervals are dangerous because of the risk of inducing tolerance. Vaccinations were given subcutaneously (s.c.) in the groin. In addition, dogs received oral meloxicam (initial dose 0.2 mg/kg body weight followed by daily 0.1 mg/kg body weight), a COX-2 inhibitor similar to piroxicam, as piroxicam is not registered for use in dogs in the Netherlands.

The CVx1 vaccine was composed of 500 μg recombinant fusion protein TRXtr-dogVimentin (TRXtr-dVim) (canis lupus familiaris, NCBI ref seq NM_001287023.1) adjuvanted with 375 μg phosphorothioate-stabilized CpG 2006 oligonucleotide (5′-T*C*G*-T*C*G-T*T*T*-T*G*T*-C*G*T*-T*T*T*-G*T*C*-G*T*T*-3′; Eurogentec, Seraing, Belgium) and 10% Montanide Gel 01PR (36067D, Seppic, Paris, France, final concentration Montanide gel 5%). Maintenance vaccinations were given without CpG. The vaccine was dosed according to body weight, with dogs >25 kg receiving the full dose of 500 μg, dogs 10–25 kg half the dose and dogs <10 kg one third of the initial dose (Table S1). Further details are available in the Supplementary Materials.

### 2.2. Immune Response Measurement

Tissue morphology of canine paraffin UC tissues obtained from the Veterinary Pathology Diagnostic Centre of Utrecht University was visualized using hematoxylin and eosin (HE) staining. These tissues were sampled from other dogs than those included in the clinical trial. Immunohistochemistry (IHC) was used to evaluate vimentin expression, according to previously published protocols [14] with minor modifications. Briefly, canine UC tissue sections (5 μm) were deparaffinized, blocked for endogenous peroxidase activity, and antigen retrieval was performed with citrate buffer. Sections were blocked with 5% bovine serum albumin (BSA) and stained with primary mouse monoclonal anti-vimentin antibody (E-5, Santa Cruz Biotechnology, sc-373717, 1:500) overnight at 4 °C. Secondary antibodies used were anti-mouse biotinylated antibody (Dako, E0433) and Streptavidin-HRP (Dako, Glostrup, Denmark, P0397). Tissues were color-developed with 3,3′-diaminobenzidine tetrahydrochloride hydrate (DAB, Sigma-Aldrich, St.Louis, MS, USA, Cat. D5637), counterstained with Mayer’s hematoxylin (VWR Chemicals, Amsterdam, The Netherlands, Cat. 10047105), dehydrated and mounted with Quick-D mounting medium (Klinipath, Duiven, The Netherlands, Cat. 7280). Images were taken with an Olympus BX50 microscope with a 10× objective and equipped with a CMEX DC 5000C camera. Further details are available in the Supplementary Material.

Blood samples were taken at start of this study, prior to vaccination, and at each follow-up visit. Blood was stored overnight at 4 °C to coagulate, and the next day, the samples were centrifuged at 4650 rcf for 10 min in a microcentrifuge to collect the serum. Serum samples were stored at −20 °C. Indirect ELISA was performed to determine total anti-dogVim antibody levels, as previously described [14].

#### 2.2.1. Anti-eVim Antibody ELISA

Canine serum samples were stored at −20 °C at the VRC until collected and transported to AG for analysis. Volumes used per well in ELISA were 50 µL, unless indicated otherwise. Firstly, 96-well ELISA plates (F96 Maxisorp, Nunc A/S, Roskilde, Denmark) were coated with 4 µg/mL recombinant dog Vimentin or mouseVimentin protein and then blocked with 4% milk/PBS (100 µL/well) (sc-2325, Santa Cruz Biotechnology, Dallas, TX, USA), both for 1 h at 37 °C. After a single wash with PBS (B. Braun Medical, Oss, The Netherlands) for 1 min, the plates were incubated with serum of TRXtr-dogVim-vaccinated dogs for 45 min at 37 °C, diluted to 1:10 in 100% horse serum, which was further diluted to 1:50, 1:100, followed by 1:300, 1:900, 1:2700, 1:8100 and 1:24,300 in 50% Rosetta Gami extract containing 100 μg/mL TRXtr-EDB protein to reduce non-specific binding of the serum. Thereafter, plates were incubated with biotinylated polyclonal goat anti-canine IgG (6070-08, Southern Biotech, Birmingham, AL, USA) for 45 min at 37 °C and streptavidin–horseradish peroxidase (P0397, Dako Cytomation, Glostrup, Denmark) for 30 min, both diluted to 1:2000 in 0.01% PBS-T at 37 °C. After each incubation step, plates were washed four times with 0.5× PBS. HRP activity was detected with TMB substrate (T-8665 or T0440, Sigma-Aldrich), and absorbance was measured at 405 nm after 15 min using a Tecan microplate reader (Tecan Sunrise, Männedorf, Switzerland).

To block background binding in ELISA, Rosetta gami DE3 extract for use in ELISA was produced from uninduced pET21a-TRX-transformed overnight cultures. Bacteria were harvested at 4500 rpm, 10 min, 4 °C (Rotina 420R, Hettich, Westphalia, Germany), and washed 3 times with PBS. The pellet (originating from 200 mL overnight culture) was resuspended in 10 mL 0.5 M urea and sonicated for 15 cycles, 20 s ‘on’ and 30 s ‘off’ (amplitude 22–26 microns, Soniprep 150 MSE). Bacterial lysates were centrifuged at 4500 rpm, 10 min, 4 °C (Rotina 420R, Hettich, Westphalia, Germany), and supernatants were saved at −20 °C until use.

#### 2.2.2. Immunofluorescence

Immunofluorescent staining of human (HMEC-1) and mouse (SVEC) endothelial cells was performed on cells grown in 96-well plates. Briefly, cells were fixated with 1% paraformaldehyde, permeabilized with 0.1% Triton-X100 and stained with pooled dog serum and monoclonal mouse anti-vimentin antibodies simultaneously. Western blotting was performed on whole cell lysates, prepared with RIPA buffer, of HMEC-1 and SVEC. Blots were probed with pooled dog serum and monoclonal mouse anti-vimentin antibodies simultaneously. Further details are available in the Supplementary Material.

### 2.3. Patient Monitoring and Evaluation

**Figure 2:**
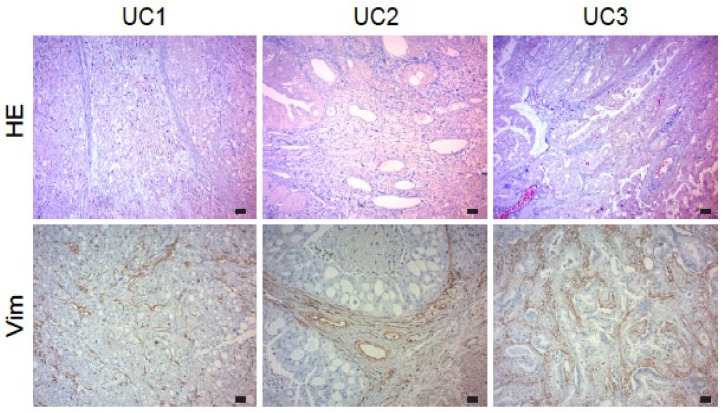
Vimentin is expressed in the tumor vasculature of UC. Hematoxylin/eosin (HE) staining of three different UC tissue samples (**upper row**). The same samples were stained with mouse monoclonal anti-vimentin antibody (**lower row**) and show a vascular staining pattern for vimentin. Representative images of a panel of *n* = 10 are shown. Scale bar 35 μm.

Dogs were re-assessed at each visit according to the treatment schedule (Figure 2). At each visit, a physical examination, a blood count, creatine and ALT (alanine transaminase) and ALP (alkaline phosphatase) levels as well as other evaluations were performed according to the veterinarian’s discretion based on the consensus of the Veterinary Cooperative Oncology Group (VCOG). The dogs were evaluated by the veterinarian for clinical response according to the VCOG consensus document on response evaluation criteria for solid tumors in dogs v1.0 based on physical condition, abdominal ultrasound and X-ray of the thorax [22]. Clinical benefit was assessed by ultrasonography instead of X-ray, which has been presented as an acceptable alternative for assessment of UC response [23], as well as by overall survival. Adverse events were assessed according to the Veterinary Cooperative Oncology Group—Common Terminology Criteria for Adverse Events v2 [24].

### 2.4. Statistical Analysis

Kaplan–Meier curves were made for overall survival, and a log-rank test was applied. The level of significance was set to *p* < 0.05 *, and GraphPad Prism(R) v9 was used for the analysis, *p* < 0.01 **.

## 3. Results

### 3.1. Expression of Vimentin in Urothelial Carcinoma

Immunohistochemistry was performed to delineate the expression of vimentin in canine UC. In concordance with observations in human UC [25], we observed abundant and specific staining of vimentin in the vasculature of canine bladder cancer tissues (Figure 2), underscoring the relevance of our approach of targeting vimentin with a vaccine.

### 3.2. Study Population

Twenty-five dogs meeting the eligibility criteria were enrolled during a time period of 18 months. One dog had a protocol violation, receiving photodynamic therapy two weeks after the 4th vaccination, and four dogs were lost to follow-up because of no-show: one after one vaccination, one after two vaccinations and two after four vaccinations. In total, twenty dogs that completed the induction vaccinations and had a first follow-up visit at least 2 months after the last vaccination were eligible for evaluation. A summary of subject characteristics compared to the control study population [21] is presented in Table 1.

### 3.3. Anti-eVim Antibody Response

All dogs developed antibody responses against extracellular vimentin (eVim), immediately after the first vaccination, which increased following the three additional biweekly induction vaccinations (Figure 1B). In one dog, the fourth injection of the induction vaccination was skipped due to grade 2 injection site reactions. However, this dog had already developed high titers against eVim after three vaccinations. Anti-eVim antibody titers generally waned over time, showing the need for subsequent maintenance vaccinations every two months (Figure S2). The induced antibody response was independent of gender (Figure 1C) and body-weight-defined vaccine dosing (Figure 1D).

### 3.4. Clinical Response Data

**Figure 3:**
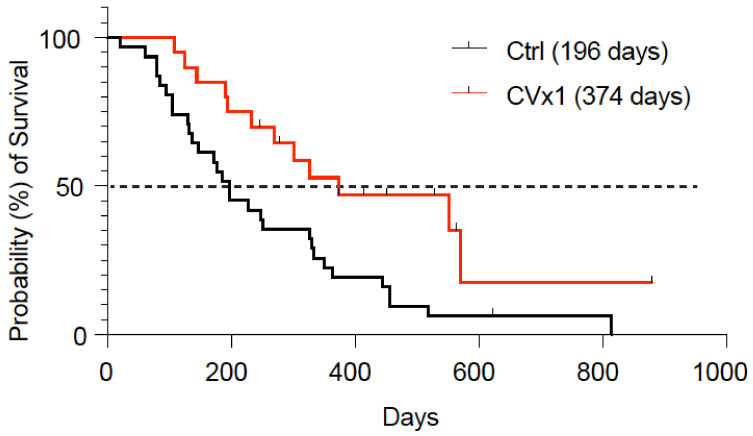
Clinical response in dogs vaccinated with CVx1. Kaplan–Meier curve of probability (%) of survival of the CVx1 vaccine (red line) compared to the historical control group (Ctrl; black line) of [21]. The dotted line marks the median overall survival.

All but one dog showed clinical benefit, defined as complete remission, partial remission or stable disease (Table 2; CVx1/meloxicam; Table S2). At the start of therapy, all dogs had T2 tumors, with marker lesions averaging 4.4 cm (length + largest diameter, range 0.9–12.3 cm); one dog had regional lymph node metastasis and one dog had regional lymph node and liver metastasis. Two dogs achieved a complete remission, two dogs showed a partial regression of the tumor and one dog showed progressive disease after 6 weeks. The other 15 dogs showed stable disease for a period longer than 8 weeks. Median progression-free interval (PFI) and overall survival (OS) were 257 and 374 days, respectively (Table 2). No statistically significant differences in median overall survival were observed for sex or weight-dependent dosing. The Kaplan–Meier curve showed a median OS of 374 days for the CVx1 treated group versus 196 days reported for the historical control group (*p* < 0.001; Figure 3). The mean durations of complete response (CR), partial response (PR) and stable disease (SD), including the ranges, were 465 (257–672), 83 (70–95) and 231 (106–563) days, respectively. Twelve dogs were euthanized during follow-up: eight because of local progressive disease and four for non-bladder-cancer-related reasons (one renal failure, one gastro-intestinal problem, one osteosarcoma in shoulder and one neural problem). At the time of evaluation (July 2022), follow-up was minimally 6 to 28 months: eight dogs were alive (four with stable and four with progressive disease). None of the 20 dogs developed evidence for regional lymph node metastasis; one dog developed distant metastases in the lung.

### 3.5. Adverse Events

Adverse events were limited to grade 1 and 2 local injection site reactions and vaccination-related clinical signs, such as nausea and fever, observed in 19 out of 20 dogs (Table 3). Adverse events occurred more frequently during the induction vaccinations (in 17 of 20 dogs) than during maintenance (in 9 of 20 dogs). Notably, the injection site reactions only required the postponement of (and a decision for a lower dose at subsequent vaccinations) treatment in one dog and skipping of the fourth induction vaccination in another dog. All adverse events were temporary and resolved spontaneously; in five cases, oral antibiotics were applied to treat an ulceration at the injection site. None of the dogs were hospitalized for adverse events, yet one dog was withdrawn from this study by the owner after two vaccinations with grade 2 injection site events.

### 3.6. Reactivity of CVx1 Sera with Mouse and Human Vimentin

**Figure 4:**
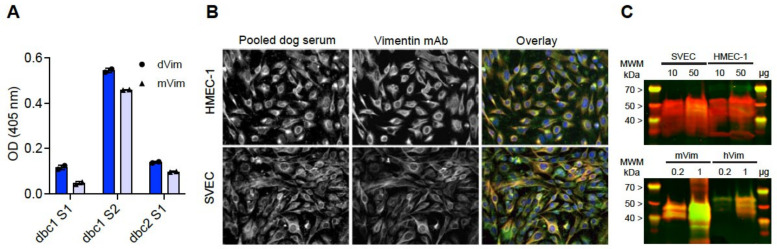
Sera of dogs vaccinated with CVx1 react with both murine and human vimentin. (**A**) Anti-eVim ELISA showing reactivity of the serum of CVx1-vaccinated dog (dbc1 and dbc2) blood samples S1, S2 with dVim (dog vimentin; dark blue bars) and mVim (mouse vimentin; light blue bars). (**B**) Immunofluorescence of human (HMEC-1) and mouse (SVEC) endothelial cells, stained with pooled dog serum (green) and mouse monoclonal anti-vimentin antibody (red). Single color channels are shown in black/white. (**C**) Western blot analysis of mouse and human endothelial cell lysates (**top**) and recombinant vimentin protein (~55 kDa) (**bottom**) with pooled dog serum (green) and mouse monoclonal anti-vimentin antibody (red). Original blots are shown in Figure S4B,C. Overlapping protein detection is visible as yellow at the expected molecular weight ~55 kD.

Vimentin is highly conserved across species (Figure S3), and the sera of CVx1-vaccinated dogs were readily reactive with recombinant dog and mouse vimentin protein in ELISA (Figure 4A). To further validate whether the vaccination induced antibody reactions with vimentin in a cellular context, we performed immunofluorescence staining and Western blot analysis. Figure 4B shows overlapping staining of filamentous vimentin (pooled dog serum in green, mouse anti-vimentin mAb in red) in both human (HMEC-1) and mouse (SVEC) cell lines. The antisera of CVx1-vaccinated dogs were also clearly reactive with recombinant mouse and human vimentin on the Western blot (Figure 4C). In addition, specific detection of vimentin at the expected molecular weight (~55 kDa) in whole cell lysates is seen after probing membranes with both dog serum (green) and mouse anti-vimentin mAb (red) (Figure 4C; Figure S4).

## 4. Discussion

In this study, we show that vaccination against extracellular vimentin (eVim) with the CVx1 vaccine extends the lifespan of dogs with urothelial carcinoma of the bladder that are treated with COX-2 inhibition, as compared to historical controls treated with carboplatin and a COX-2 inhibitor [21]. Vaccination with CVx1 was well tolerated, induced anti-eVim antibodies and did not show any antibody-induced systemic side effects. As the study population was not a homogeneously inbred population, the differences in responses and clinical benefit observed were expected.

We have previously validated the strong overexpression of vimentin in the tumor vasculature of solid tumors [15] and demonstrated that vimentin is excreted by the tumor vasculature to support angiogenesis and to escape anti-tumor immunity. In preclinical models, the targeting of vimentin with antibody therapy or vaccination significantly inhibited tumor growth [14]. We confirmed the overexpression of vimentin in the tumor vasculature of a panel of dog UC with immunohistochemistry, underscoring the translational relevance of the CVx1 anti-eVim vaccine from mice to dogs. Interestingly, very recently vimentin expression in tumor stroma was postulated as a potential prognostic marker for canine gastric cancer [33], supporting our observations. Authors should discuss the results and how they can be interpreted from the perspective of previous studies and of the working hypotheses. The findings and their implications should be discussed in the broadest context possible. Future research directions may also be highlighted.

Vaccination has multiple advantages over treatment with a monoclonal antibody. Firstly, vaccination induces a polyclonal antibody response that can be assumed to neutralize the target better than a monoclonal antibody. Secondly, autologous antibodies penetrate the tumor tissue more easily, for example because a mature plasma cell can eventually reside inside the tumor tissue. Thirdly, vaccination is less invasive and can be provided in peripheral centers without hospitalization or even by general practitioners. Fourthly, vaccination requires the production and application of only small protein quantities compared to monoclonal antibody therapy. The effectiveness of the anti-eVim antibodies is expected at three different levels. Antibodies bound to the target in the tumor vasculature induce antibody-dependent cell cytotoxicity (ADCC) in endothelial cells, executed by Fc-receptor-expressing white blood cells. In previous studies to another target, this was shown to happen in the form of ‘frustrated phagocytosis’ [34]. Also, bound antibodies are expected to induce the complement system, leading to the generation of the membrane attack complex and the subsequent lysis of endothelial cells. This was not proven to occur. In addition, induced antibodies have a functional effect by blocking the function of eVim. As shown before, this results in the blockade of endothelial mobility and hence the prevention of vascular sprouting, as well as reversal of the immunosuppressive function of eVim, resulting in the enhanced infiltration of anti-tumor immune cells [14]. Finally, it has been shown that the inhibition of angiogenesis generally improves leukocyte infiltration through mechanisms which increase the endothelial cell adhesiveness [35,36,37]. This renders the combination therapy of CVx1 with immune checkpoint blockade or CAR-T-cell therapy highly warranted.

The antibody response to the target antigen vimentin is based on the conjugate vaccine technology, i.e., the bacterial sequences induce a normal T-cell response that eventually helps the auto-reactive B cells to proliferate and produce antibodies. In principle, this procedure does not induce T cells against the target antigen. In a previous study by Van Loon et al., it was shown that this is indeed true for CD8 cells, while a moderate CD4 response was noted [17]. Therefore, we think that a similar response was generated in the dogs in our study.

Canine UC is a disease that closely resembles human bladder cancer, both histologically and clinically [19,20]. The treatment options for dogs with UC are limited. Like in humans, various chemotherapeutic agents are available, such as vinblastine, cisplatin, carboplatin, doxorubicin, gemcitabine and mitoxantrone, which are often applied in combination with COX-2 inhibitors, such as piroxicam or meloxicam [38]. Recently, targeted therapies have also been studied, such as folate-tubulysin, which targets tumors with high folate receptor expression, and toceranib, which prevents tyrosine kinase receptor activation [39,40]. All these treatments show median survival times (OS) ranging from 41 to 299 days [38]. Yet, its frequent and dose-limiting renal toxicity makes this combination unacceptable to be recommended for routine use. COX-2 inhibitors alone offer a useful palliative treatment, are generally well tolerated and offer a small advantage. The OS reported for single piroxicam treatment ranges from 181 to 244 days [29,41,42]. COX-2 inhibition is currently the standard treatment for UC and other canine cancers when chemotherapy is not possible [43].

Consequently, in this study, the CVx1 anti-eVim vaccine was tested in combination with meloxicam, which, unlike piroxicam, is registered for use in dogs in the Netherlands. Although this COX-2 inhibitor may suppress immunity, this did not prevent the induction of antibody responses against eVim, as all vaccinated dogs showed efficient induction of circulating antibodies against the vaccine target. Interestingly, anti-eVim antibodies were detected already 2 weeks after the first vaccination, and the antibody titers increased further after each booster. Anti-eVim antibody titers waned over time, similarly to what was reported in mice [14]. With the maintenance vaccinations at 2-month intervals, the antibody titers could be maintained at post-induction vaccination values for the duration of this study. Although the dog serum pool showed signs of multi-reactivity with cellular proteins in Western blot analysis and the immunostaining of cells (which is to be expected given its polyclonal nature), the clear and abundant detection of vimentin was observed. Moreover, clear intracellular filamentous staining was observed with the sera on endothelial cells, demonstrating that the vaccine-induced antibody response is relevant for vimentin in a physiological context.

The median OS of 374 days seen in our study compares favorably to any other drug treatment for canine bladder cancer (Table 2) and offers a significant clinical benefit over piroxicam alone. However, a limitation of the current study is the lack of a parallel control group. The safety and efficacy of the CVx1 vaccine combined with meloxicam were only compared to a historical control group treated with carboplatin and piroxicam 20 and not to piroxicam treatment alone, as no Kaplan–Meier data from this study were available [29]. Nevertheless, showing the benefit over treatment with chemotherapy plus COX-2 inhibition may be even more relevant and urges translational development. Another limitation of this study is that we did not include a vaccine-only group, as our ethical approval did not allow this because of the small clinical benefit of piroxicam. However, the major clinical benefit observed must be attributed to the vaccine.

All client-owned dogs treated in this study showed a clinical benefit, while two dogs showed a complete remission. Although most dogs ultimately developed progressive disease, the cancer was detected only locally, and only one of the dogs (3.7%) had developed a lung metastasis, as detected by X-ray imaging. Interestingly, from previously published work, it is known that 26–42% of dogs present with nodal and 50–58% with distant metastases at the time of death [27,42]. Considering the fact that we almost tripled the overall survival and distant metastasis is therefore expected to increase, the low number of detected metastases in this study is exceptionally noteworthy.

Pertaining to the tolerability of the treatment regimen tested in this study, no unacceptable adverse events were recorded. In one dog, multiple necrotic areas appeared in its primary tumor after the second vaccination [14]. This eventually resulted in severe hematuria, which required the resection of the tumor after the fourth induction vaccination, and post-operative H&E staining of tumor tissue showed clear intralesional presence of immune cells [14]. Thereafter, the dog was tumor-free until 676 days after the start of treatment. The recurring tumor was surgically removed again, and maintenance vaccinations continued also beyond the end of this study (July 2022). A second dog with a history of recurrent bladder cancer showed a complete response following induction vaccination. Sometime after surgery for reconstruction of the ureter, the dog presented with recurrent local disease. The dog continued on vaccination treatment and developed adequate anti-eVim antibody titers again, resulting in stable disease [14]. Eventually, the disease progressed, and the dog was euthanized 553 days after the first vaccination. Both dogs that underwent surgery did not show any signs of impaired/delayed wound healing, which was in accordance with previous observations in mice [14].

The CVx1 vaccine consists of the recombinant vaccine protein and a very potent adjuvant (Montanide gel/CpG), which is necessary to break the immunological self-tolerance to be able to induce an antibody response against the self-antigen extracellular vimentin. Montanide Gel 01PR alone has been tested in dogs previously and is associated with adverse effects, such as induration at the injection site, fever and fatigue [44]. In the current study, these adverse effects were seen as well, yet these were only temporary, self-limiting and minor to moderate in nature. The adverse events, however, might not solely be attributed to the Montanide gel but might also be caused by the toll-like receptor 9 (TLR-9) agonist CpG 2006 or a combination of both. As maintenance vaccinations were performed without CpG oligo and injection site reactions were still, but less frequently, observed, they were most likely caused by the Montanide gel adjuvant. For the translation of the vaccine for human use, these data are less relevant, as the human vaccine formulation will be with an adjuvant consisting of a combination of the squalene-based adjuvant Montanide ISA 720 and the CpG 7909 oligo, specific for the human TLR-9. This adjuvant has been clinically tested before [45].

In the current study, we observed repeated clinical anti-tumor responses to vaccination with CVx1. In cancer therapy this is very unusual, as in most cases, if not all, resistance to therapy occurs. We favor the view that our observations are the result of targeting the non-neoplastic tumor vasculature and not the tumor cells themselves, as the vimentin-excreting tumor endothelial cells are genetically more stable than highly mutating tumor cells and do not easily mutate into resistant variants. If this is true, it would be the first observation demonstrating a lack of resistance, since other drugs that are expected to target the vasculature also have effects on tumor cells (e.g., tyrosine kinase inhibitors) or are targeted towards tumor-produced growth factors (e.g., bevacizumab, aflibercept).

## 5. Conclusions

The overexpression and secretion of vimentin by endothelial cells in solid tumors renders this protein an excellent target for therapeutic intervention in cancer therapy. Based on proven efficacy and safety in mice, we developed CVx1, a vaccine against extracellular vimentin (eVim), for the treatment of dogs with UC of the bladder. CVx1 treatment in combination with standard COX-2 inhibition was demonstrated to be effective and safe for the treatment of client-owned dogs with spontaneous UC of the bladder and allows for long-term treatment. CVx1 plus COX-2 inhibitors compared favorably to cisplatin plus COX-2 inhibitors in a historic survival analysis. Importantly, anti-vimentin immunity could be induced in all dogs, and recurrent vaccinations were able to sustain hyperimmunity. These results support the translation of the CVx1 vaccine into the clinic for the treatment of human patients with bladder cancer and possibly other solid tumors. A phase 1-2a clinical trial in humans with UC of the bladder of CVx1 combined with COX-2 inhibition with or without immune checkpoint blockade is being scheduled.

## 6. Patents

PCT/NL2017/050526, Embryonic angiogenesis markers and diagnostic and therapeutic strategies based thereon. Arjan W. Griffioen, Elisabeth J. M. Huijbers and Patrycja Nowak-Sliwinska.

## Figures and Tables

**Table 1:** Study population characteristics. Control (Ctrl), male (M), female (F), therapy (Tx), tumor stage (T), movable enlarged lymph nodes (N) and detectable metastasis (M).

	Dogs (*n*)	Gender, *n* (%)		Breed, *n* (%)		Weight, Median	Age, Average	Prior Tx *	Stage, *n* (%)					
		M	F	Single	Mixed	kg (range)	year (range)	*n* (%)	T_2_N_0_M_0_	T_2_N_1_M_0_	T_2_N_0_M_1_	T_2_N_2_M_1_	T_3_N_0_M_0_	T_3_N_1_M_1_
Study	20	8 (40%)	12 (60%)	17 (85%)	3 (15%)	18.8 (4.4–72)	11 (7–14)	5 (25%)	15 (75%)	2 (10%)	2 (10%)	1 (5%)	0 (0%)	0 (0%)
Ctrl	31	12 (39%)	19 (61%)	25 (81%)	6 (19%)	14.4 (3.6–47)	11 (6–15)	6 (19%)	24 (75%)	2 (6%)	2 (6%)	0 (0%)	1 (3%)	2 (6%)

* Prior treatment: surgery, photodynamic therapy, cisplatin, mitroxantrone, doxorubicin, actinomycin D, piroxicam.

**Table 2:** Comparison of current study results with historical studies reported for chemotherapy/piroxicam combination therapy and piroxicam alone of UC in dogs. Clinical response criteria per VCOG consensus [22]. N1 = movable enlarged lymph nodes on same side of body, N2 = movable enlarged lymph nodes on opposite side of body or bilateral, M1 = detectable distant metastasis, complete response (CR), partial response (PR), stable disease (SD), progressive disease (PD), progression-free interval (PFI) and overall survival (OS).

	Dogs	N1, N2/M1	CR	PR	SD	PD	PFI	OS	Refs.
	Total/Evaluable or Tumor Response	Any Metastasis, % of Total							
**Drugs**	**(#)**	**(%)**	**(%)**	**(%)**	**(%)**	**(%)**	**days**	**days**	
**Current study**									
CVx1/meloxicam	**25/20**	**5/5/10**	**10**	**10**	**75**	**5**	**257**	**374**	
**Randomized trials**									
Vinblastine/piroxicam	27/26	0/4/4	0	58	33	8	199	299	[26]
Cisplatin/piroxicam	14/14	28/14/43	14	57	28	0	124	246	[27]
Mitoxantrone/piroxicam	26/NA	8/NA/8	0	8	69	23	106	247	[28]
Carboplatin/piroxicam	24/NA	29/NA/29	0	13	54	33	73	263	[28]
**Single-arm trials**									
Piroxicam	34/34	9/15/24	2	4	18	10	NA	181	[29]
Mitoxantrone/piroxicam	55/48	NA/NA/11	2	33	46	19	194	291	[30]
Carboplatin/piroxicam	31/29	13/13/19	0	38	45	17	NA	161	[21]
Doxorubicin/piroxicam	34/23	NA/NA/NA	0	9	60	30	103	168	[31]
Gemcitabine/piroxicam	38/37	11/3/11	5	22	51	22	NA	230	[32]

**Table 3:** Adverse events.

	Dogs *, *n* = 20 (%)			Vaccinations, *n* = 172 (%)			
				Initial, *n* =79		Maintenance, *n* = 66	
	Grade 1	Grade 2	Grade 3–5	Grade 1	Grade 2	Grade 1	Grade 2
Administration site conditions							
Injection site reactions	9 (45%)	7 (35%)	0 (0%)	27 (34%)	8 (10%)	10 (15%)	5 (8%)
Lameness local extremity	5 (25%)	1 (5%)	0 (0%)	2 (3%)	0 (0%)	0 (0%)	0 (0%)
Constitutional clinical signs							
Lethargy	3 (15%)	5 (25%)	0 (0%)	7 (9%)	6 (6%)	0 (0%)	1 (2%)
Anorexia	1 (5%)	0 (0%)	0 (0%)	0 (0%)	0 (0%)	1 (2%)	0 (0%)
Nausea/vomiting	5 (25%)	0 (0%)	0 (0%)	4 (5%)	0 (0%)	3 (5%)	0 (0%)
Fever	2 (10%)	0 (0%)	0 (0%)	4 (5%)	0 (0%)	0 (0%)	0 (0%)
Diarrhea	2 (10%)	0 (0%)	0 (0%)	1 (1%)	0 (0%)	1 (2%)	0 (0%)
Any *	11 (55%)	8 (40%)	0 (0%)	12 (60%)	5 (25%)	5 (25%)	4 (20%)

* The highest grade of any event is scored for each dog.

## Data Availability

The data presented in this study are available on request from the corresponding author.

## Supplementary Materials

The following supporting information can be downloaded at https://www.mdpi.com/article/10.3390/cancers15153958/s1, Figure S1: Illustration of the immunological response of vaccination at the cellular level, Figure S2: Anti-Vim antibody response over time, Figure S3: Sequence alignment of vimentin, Figure S4: Reactivity of canine sera with murine and human vimentin, Table S1: Vaccine composition, Table S2: Patient data. Censored: 1 = euthanized, 0 = alive in follow.

## References

[1] Sung H., Ferlay J., Siegel R.L., Laversanne M., Soerjomataram I., Jemal A., Bray F. (2021). Global Cancer Statistics 2020: GLOBOCAN Estimates of Incidence and Mortality Worldwide for 36 Cancers in 185 Countries. CA Cancer J. Clin..

[2] Richters A., Aben K.K.H., Kiemeney L.A.L.M. (2020). The global burden of urinary bladder cancer: An update. World J. Urol..

[3] Sievert K.D., Amend B., Nagele U., Schilling D., Bedke J., Horstmann M., Henenlotter J., Kruck S., Stenzl A. (2009). Economic aspects of bladder cancer: What are the benefits and costs?. World J. Urol..

[4] Leal J., Luengo-Fernandez R., Sullivan R., Witjes J.A. (2016). Economic Burden of Bladder Cancer Across the European Union. Eur. Urol..

[5] Svatek R.S., Hollenbeck B.K., Holmäng S., Lee R., Kim S.P., Stenzl A., Lotan Y. (2014). The economics of bladder cancer: Costs and considerations of caring for this disease. Eur. Urol..

[6] Sanli O., Dobruch J., Knowles M.A., Burger M., Alemozaffar M., Nielsen M.E., Lotan Y. (2017). Bladder cancer. Nat. Rev. Dis. Prim..

[7] Witjes J.A., Bruins H.M., Cathomas R., Compérat E.M., Cowan N.C., Gakis G., Hernández V., Linares Espinós E., Lorch A., Neuzillet Y. (2021). European Association of Urology Guidelines on Muscle-invasive and Metastatic Bladder Cancer: Summary of the 2020 Guidelines. Eur. Urol..

[8] Saginala K., Barsouk A., Aluru J.S., Rawla P., Padala S.A., Basouk A. (2020). Epidemiology of Bladder Cancer. Med. Sci..

[9] Mukherjee N., Svatek R.S., Mansour A.M. (2018). Role of immunotherapy in bacillus Calmette–Guérin-unresponsive non–muscle invasive bladder cancer. Urol. Oncol. Semin. Orig. Investig..

[10] Patel V.G., Oh W.K., Galsky M.D. (2020). Treatment of muscle-invasive and advanced bladder cancer in 2020. CA Cancer J. Clin..

[11] Roviello G., Catalano M., Santi R., Palmieri V.E., Vannini G., Galli I.C., Buttitta E., Villari D., Rossi V., Nesi G. (2021). Immune checkpoint inhibitors in urothelial bladder cancer: State of the art and future perspectives. Cancers.

[12] Bergman P.J., Camps-Palau M.A., McKnight J.A., Leibman N.F., Craft D.M., Leung C., Liao J., Riviere I., Sadelain M., Hohenhaus A.E. (2006). Development of a xenogeneic DNA vaccine program for canine malignant melanoma at the Animal Medical Center. Vaccine.

[13] Bow S., Guth A., Vail D.M., Thamm D.H., Liptak. J.M. (2020). Cancer Immunotherapy. Withrow and MacEwen’s Small Animal Clinical Oncology.

[14] Van Beijnum J.R., Huijbers E.J.M., van Loon K., Blanas A., Akbari P., Roos A., Wong T.J., Denisov S.S., Hackeng T.M., Jimenez C.R. (2022). Extracellular vimentin mimics VEGF and is a target for anti-angiogenic immunotherapy. Nat. Commun..

[15] Van Beijnum J.R., Dings R.P., van der Linden E., Zwaans B.M.M., Ramaekers F.C.S., Mayo K.H., Griffioen A.W. (2006). Gene expression of tumor angiogenesis dissected: Specific targeting of colon cancer angiogenic vasculature. Blood.

[16] Huijbers E.J.M., van Beijnum J.R., Lê C.T., Langman S., Nowak-Sliwinska P., Mayo K.H., Griffioen A.W. (2018). An improved conjugate vaccine technology; induction of antibody responses to the tumor vasculature. Vaccine.

[17] Van Loon K., Huijbers E.J.M., de Haan J.D., Griffioen A.W. (2022). Cancer Vaccination against Extracellular Vimentin Efficiently Adjuvanted with Montanide ISA 720/CpG. Cancers.

[18] Patrick D.J., Fitzgerald S.D., Sesterhenn I.A., Davis C.J., Kiupel M. (2006). Classification of Canine Urinary Bladder Urothelial Tumours Based on the World Health Organization/International Society of Urological Pathology Consensus Classification. J. Comp. Pathol..

[19] Knapp D.W., Dhawan D., Ramos-Vara J.A., Ratliff T.L., Cresswell G.M., Utturkar S., Sommer B.C., Fulkerson C.M., Hahn N.M. (2019). Naturally-Occurring Invasive Urothelial Carcinoma in Dogs, a Unique Model to Drive Advances in Managing Muscle Invasive Bladder Cancer in Humans. Front. Oncol..

[20] Dow S. (2020). A Role for Dogs in Advancing Cancer Immunotherapy Research. Front. Immunol..

[21] Boria P.A., Glickman N.W., Schmidt B.R., Widmer W.R., Mutsaers A.J., Adams L.G., Snyder P.W., DiBernardi L., De Gortari A.E., Bonney P.L. (2005). Carboplatin and piroxicam therapy in 31 dogs with transitional cell carcinoma of the urinary bladder. Vet. Comp. Oncol..

[22] Nguyen S.M., Thamm D.H., Vail D.M., London C.A. (2015). Response evaluation criteria for solid tumours in dogs (v1.0): A Veterinary Cooperative Oncology Group (VCOG) consensus document. Vet. Comp. Oncol..

[23] Honkisz S.I., Naughton J.F., Weng H.Y., Fourez L.M., Knapp D.W. (2018). Evaluation of two-dimensional ultrasonography and computed tomography in the mapping and measuring of canine urinary bladder tumors. Vet. J..

[24] LeBlanc A.K., Atherton M., Bentley R.T., Boudreau C.E., Burton J.H., Curran K.M., Dow S., Giuffrida M.A., Kellihan H.B., Mason N.J. (2021). Veterinary Cooperative Oncology Group—Common Terminology Criteria for Adverse Events (VCOG-CTCAE v2) following investigational therapy in dogs and cats. Vet. Comp. Oncol..

[25] Rahmani A.H., Babiker A.Y., Alwanian W.M., Elsiddig S.A., Faragalla H.E., Aly S.M. (2015). Association of cytokeratin and vimentin protein in the genesis of transitional cell carcinoma of urinary bladder patients. Dis. Markers.

[26] Knapp D.W., Ruple-Czerniak A., Ramos-Vara J.A., Naughton J.F., Fulkerson C.M., Honkisz S.I. (2016). A nonselective cyclooxygenase inhibitor enhances the activity of vinblastine in a naturally-occurring canine model of invasive urothelial carcinoma. Bladder Cancer.

[27] Knapp D.W., Glickman N.W., Widmer W.R., DeNicola D.B., Adams L.G., Kuczek T., Bonney P.L., DeGortari A.E., Han C., Glickman L.T. (2000). Cisplatin versus cisplatin combined with piroxicam in a canine model of human invasive urinary bladder cancer. Cancer Chemother. Pharmacol..

[28] Allstadt S.D., Rodriguez C.O., Boostrom B., Rebhun R.B., Skorupski K.A. (2015). Randomized Phase III Trial of Piroxicam in Combination with Mitoxantrone or Carboplatin for First-Line Treatment of Urogenital Tract Transitional Cell Carcinoma in Dogs. J. Vet. Intern. Med..

[29] Knapp D.W., Richardson R.C., Chan T.C., Bottoms G.D., Widmer W.R., DeNicola D.B., Teclaw R., Bonney P.L., Kuczek T. (1994). Piroxicam therapy in 34 dogs with transitional cell carcinoma of the urinary bladder. J. Vet. Intern. Med..

[30] Henry C.J., McCaw D.L., Turnquist S.E., Tyler J.W., Bravo L., Sheafor S., Straw R.C., Dernell W.S., Madewell B.R., Jorgensen L. (2003). Clinical evaluation of mitoxantrone and piroxicam in a canine model of human invasive urinary bladder carcinoma. Clin. Cancer Res..

[31] Robat C., Burton J., Thamm D., Vail D. (2013). Retrospective evaluation of doxorubicin-piroxicam combination for the treatment of transitional cell carcinoma in dogs. J. Small Anim. Pract..

[32] Marconato L., Zini E., Lindner D., Suslak-Brown L., Nelson V., Jeglum A.K. (2011). Toxic effects and antitumor response of gemcitabine in combination with piroxicam treatment in dogs with transitional cell carcinoma of the urinary bladder. J. Am. Vet. Med. Assoc..

[33] Flores A.R., Rêma A., Mesquita J.R., Taulescu M., Seixas F., Gärtner F., Amorim I. (2022). Vimentin and Ki-67 immunolabeling in canine gastric carcinomas and their prognostic value. Vet. Pathol..

[34] Huijbers E.J.M., Ringvall M., Femel J., Kalamajski S., Lukinius A., Åbrink M., Hellamn L., Olsson A.K. (2010). Vaccination against the extra domain-B of fibronectin as a novel tumor therapy. FASEB J..

[35] Griffioen A.W., Damen C.A., Martinotti S., Blijham G.H., Groenewegen G. (1996). Endothelial intercellular adhesion molecule-1 expression is suppressed in human malignancies: The role of angiogenic factors. Cancer Res..

[36] Nowak-Sliwinska P., van Beijnum J.R., Griffioen C.J., Huinen Z.R., Sopesens N.G., Schulz R., Jenkins S.V., Dings R.P.M., Groenendijk F.H., Huijbers E.J.M. (2022). Proinflammatory activity of VEGF-targeted treatment through reversal of tumor endothelial cell anergy. Angiogenesis.

[37] Huijbers E.J.M., Khan K.A., Kerbel R.S., Griffioen A.W. (2022). Tumors resurrect an embryonic vascular program to escape immunity. Sci. Immunol..

[38] Fulkerson C.E., Knapp D.W. (2020). Tumors of the urinary system. Withrow and MacEwen’s Small Animal Clinical Oncology.

[39] Szigetvari N.M., Dhawan D., Ramos-Vara J.A., Leamon C.P., Klein P.J., Audrey Ruple A., Heng H.G., Pugh M.R., Rao S., Vlahov I.R. (2018). Phase I/II clinical trial of the targeted chemotherapeutic drug, folate-tubulysin, in dogs with naturally-occurring invasive urothelial carcinoma. Oncotarget.

[40] Lynn Gustafson T., Biller B. (2019). Use of Toceranib Phosphate in the Treatment of Canine Bladder Tumors: 37 Cases. J. Am. Anim. Hosp. Assoc..

[41] Knapp D.W., Ramos-Vara J.A., Moore G.E., Dhawan D., Bonney P.L., Young K.E. (2014). Urinary bladder cancer in dogs, a naturally occuring model for cancer biology and drug development. ILAR J..

[42] Iwasaki R., Shimosato Y., Yoshikawa R., Goto S., Yoshida K., Murakami M., Kawabe M., Sakai H., Mori T. (2019). Survival analysis in dogs with urinary transitional cell carcinoma that underwent whole-body computed tomography at diagnosis. Vet. Comp. Oncol..

[43] Burgess K.E., DeRegis C.J. (2019). Urologic Oncology. Vet. Clin. N. Am. Small Anim. Pract..

[44] Parker R., Deville S., Dupuis L., Bertrand F., Aucouturier J. (2009). Adjuvant formulation for veterinary vaccines: Montanide^TM^ Gel safety profile. Procedia Vaccinol..

[45] NIH US National Library of Medicine (2009). CpG 7909/Montanide ISA 720 with or without Cyclophosphamide in Combination Either with NY-ESO-1-derived Peptides or the NY-ESO-1 Protein for NY-ESO-1-expressing Tumors (NCT00819806). NCT00819806.

