# Supplementary material for: Vaccination against Extracellular Vimentin for Treatment of Urothelial Cancer of the Bladder in Client-Owned Dogs
Source: Cancers (Basel). 2023 Aug 3;15(15):3958. doi: 10.3390/cancers15153958 (PMC10417384; doi:10.3390/cancers15153958)
Supplement: Supplementary file 1 [file cancers-15-03958-s001.zip › cancers-2489104-supplementary.pdf]

## Supplementary data

### 1. Materials and methods

### 1.1 Vaccine composition and dosing

CVx1 consists of the vaccine protein and a potent adjuvant (Montanide gel/CpG), which is necessary to break the immunological self-tolerance against the self-antigen extracellular vimentin. The CVx1 vaccine was composed of 500µg recombinant fusion protein TRXtr-dogVimentin (TRXtr-dVim) (canis lupus familiaris, NCBI ref seq NM_001287023.1) adjuvanted with 375µg phosphorothioate stabilized CpG 2006 oligonucleotide (5′-T*C*G*-T*C*G-T*T*T*-T*G*T*-C*G*T*-T*T*T*-G*T*C*-G*T*T*-3′; Eurogentec) and 10% Montanide Gel 01PR (36067D, Seppic, Paris, France, final concentration Montanide gel 5%). Maintenance vaccinations were given without CpG. The vaccine was dosed according to body weight with dogs >25 kg receiving the full dose of 500µg, dogs 10-25 kg half the dose and dogs <10kg one third of the initial dose (Table S1).

**Table S1.** Vaccine composition

| | Body weight | TRXtr-dVim | CpG 2006 | Montanide gel 10% | Injection volume |
|---|---|---|---|---|---|
| Initial | >25 kg | 500µg | 375µg | 500µl | 1.075ml |
| | 10-25 kg | 250µg | 187.5µg | 250µl | 0.538ml |
| | <10 kg | 125µg | 125µg | 125µl | 0.300ml |
| Maintenance | >25 kg | 250µg | | 250µl | 0.500ml |
| | 10-25 kg | 250µg | | 250µl | 0.500ml |
| | <10 kg | 150µg | | 150µl | 0.300ml |

The recombinant TRXtr-dVim protein was produced in *E. coli* BL21 DE3 (Novagen; Merck Millipore, Darmstadt, Germany) and purified as previously described [1] and contained the dog (NM_001287023.1) vimentin protein coding sequence in frame with truncated TRX (TRXtr). The phosphorothioate stabilized CpG 2006 oligonucleotide has been described previously (R. Rankin et al. Antisense Nucleic acid drug development 2001) and was produced by Eurogentec with the following sequence: 5′-T*C*G*-T*C*G-T*T*T*-T*G*T*-C*G*T*-T*T*T*-G*T*C*-G*T*T*-3′. Montanide Gel 01PR was sourced from Seppic, Paris, France (article code 36067D). A concentration of 10% Montanide gel in PBS was prepared prior to mixing with the vaccine protein/CpG in the syringe at the moment of administration. The Montanide gel end concentration is 5% in the final vaccine preparation [2].

### 1.2. Immunofluorescence

Immunofluorescent staining of human (HMEC-1) and mouse (SVEC) endothelial cells was performed with pooled dog serum and monoclonal mouse anti-vimentin antibodies simultaneously.

HMEC-1 (immortalized human vascular ECs; ATCC CRL-3243) and SVEC 4-10 (mouse ECs; ATCC CRL-2181) were maintained in RPMI and DMEM cell culture medium, respectively, supplemented with 2mM glutamine (Life Technologies), 1% of antibiotics (penicillin/streptomycin, Life Technologies) and 10% NBCS (Lonza). Cells (20000/well) were plated in 0.2% gelatin-coated 96-well plates (VWR) and grown to semi-confluence overnight. Cells were fixated with 1% PFA in PBS for 20′ at RT, then permeabilized with 0.1% Triton in PBS for 10′ at RT. Cells were blocked with PBS/1%BSA for 30′ at RT. First, cells were incubated with primary antibodies reactive with both human and mouse

vimentin (E5, Santa Cruz sc373717; 1:100 in PBS/1% BSA), followed by polyclonal rabbit anti-mouse serum (DAKO Z0259; 1:200 in PBS/1%BSA) and then by TRITC conjugated swine anti-rabbit antibody (DAKO R0156; 1:200 in PBS/1%BSA). All incubations were performed in 50ul volumes for 1h at RT in the dark, followed by 3 washes with 100ul PBS. Thereafter, pooled (N=5 dogs) dog serum was applied to the cells (1:200 in PBS/1%BSA), followed by biotinylated goat anti-canine IgG (Southern Biotech, cat no. 6070-08; 1:200 in PBS/1%BSA) and Streptavidin-A488 (Life Technologies S11223; 1:500 in PBS/1%BSA) with similar incubation conditions. Finally, nuclei were stained with DAPI (Sigma). Images were aquired with a Leica DMIL microscope with fluorescence unit equipped with an FC345Fx camera, using a 20x objective. Images were analysed using Leica Application Suite v4.13.10 (Leica), and post-processed using Adobe Photoshop CS6.

**1.4 Western blot**

Western blotting was performed on whole cell lysates. Blots were probed with pooled dog serum and monoclonal mouse anti-vimentin antibodies simultaneously.

Cells (HMEC-1 and SVEC) were cultured to near confluence in standard T75 cell culture flasks (Greiner), harvested by trypsinization and pelleted. Cells were resuspended in 100µl RIPA buffer (50mM Tris-HCl pH8.0, 150mM NaCl, 0.5% SDS, 0.1% sodium deoxycholate, 1% NP-40), incubated on ice for 20′ and then spun at 20000g for 10′ at 4°C. Cleared lysate was transferred to a fresh tube. Protein concentration was measured using a micro BCA assay (Thermo Fischer Scientific). Ten to 50µg total protein lysate, or 200 to 1000ng recombinant human and mouse vimentin (Sino Biological and in-house produced [1]were loaded onto 4-12% polyacrylamide gels (Invitrogen), run alongside the Spectra Multicolor Broad Range Protein Ladder (Thermo Fischer Scientific) and transferred to a polyvinylidene difluoride membrane (Sigma). Blots were blocked with 50% blocking buffer (Rockland) in PBS, after which pooled dog serum (1:2000) and mouse monoclonal anti-vimentin antibodies (in-house; 1:5000) were added simultaneously [1]. Blots were incubated on a roller at 4°C overnight, and subsequently washed 3x10′ in PBS/0.1% Tween-20. Biotinylated goat anti-dog antibody (1:4000) was incubated in 50% blocking buffer in PBS for 1h at RT, followed by washes as above. Finally, Streptavidin IRDye 800 (LI-COR 926-32230, green; 1:10000) and donkey anti-mouse IRDye 680 (LI-COR 926-68072, red; 1:10000), were incubated in 50% blocking buffer in PBS for 1h at RT, followed by washes as above, with a final wash in PBS only. Blots were scanned with the LI-COR Odyssey CLx scanner. Images were further processed using Adobe Photoshop CS6.

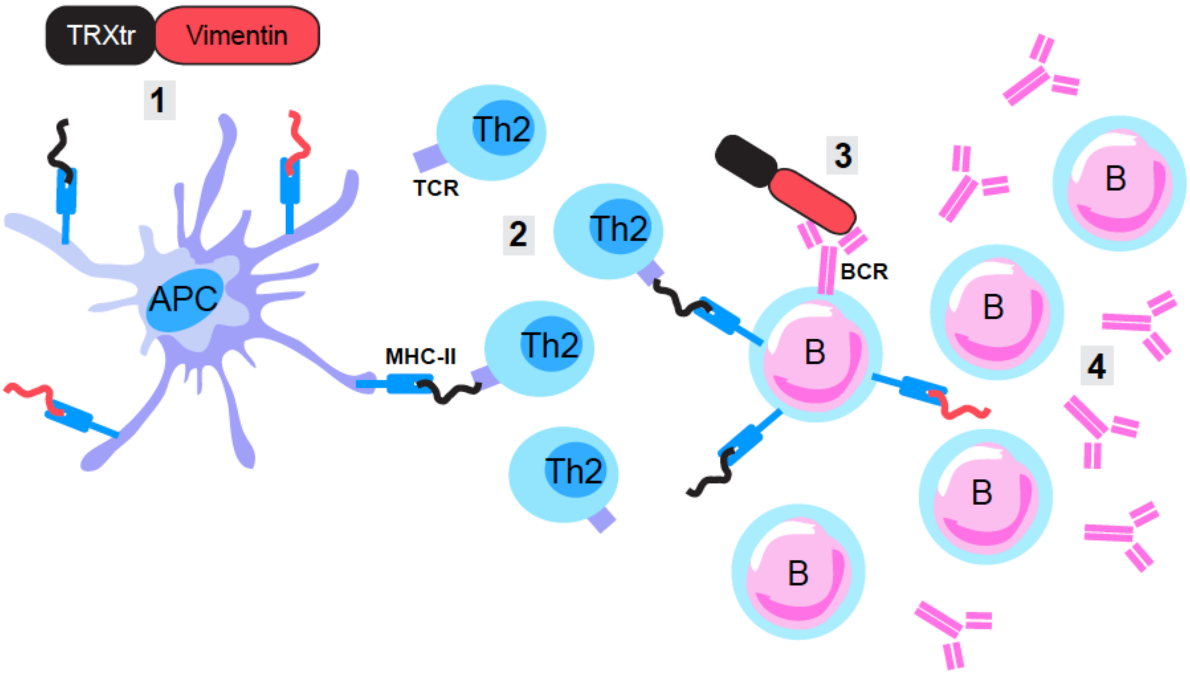


**Figure S1.** Illustration of the immunological response of vaccination at the cellular level. The vaccine protein (TRXtr-Vimentin) is injected together with a potent adjuvant. After injection it will be taken up by antigen presenting cells (APC). The APC will digest the fusion protein and present peptides of TRXtr (foreign) and vimentin (self) on major histocompatibility complex class II (MHC-II) on their surface (1). T helper 2 cells (Th2) that recognize the foreign TRXtr via their T cell receptor (TCR) will be activated (2). Self (auto)-reactive B cells (B) will recognize the self-part of the fusion protein via their B cell receptor (BCR) (3). These B cells will also present foreign- and self-peptides of the TRXtr-Vimentin fusion protein on their surface in MHC-II and by this means the TRXtr-reactive Th2 cells will be tricked to provide activation help to the self-reactive B cells. The self-reactive B cells will clonally expand and produce anti-vimentin antibodies (4).

**2. Results**

**Table S2.** Patient data. Censored: 1= euthanized, 0= alive in follow

| Dog | Race | Gender | Age (yr) | Weight (kg) | Survival (days) | Censored | Urethra | Prostate | Antibody titer S4 |
|---|---|---|---|---|---|---|---|---|---|
| 1 | Spanish Galgo | Female, sprayed | 11.9 | 26.6 | 551 | 1 | 0 | 0 | 515.8 |
| 2 | Bernesian Mountain dog | Male, castrated | 7.0 | 68.0 | 879 | 0 | 0 | 0 | 748.9 |
| 3 | Podenco | Male, castrated | 13.9 | 19.0 | 108 | 1 | 0 | 0 | 1050.0 |
| 4 | Terrier | Male, castrated | 11.2 | 23.9 | 232 | 1 | 0 | 0 | 36495.5 |
| 5 | Boomer | Female, sprayed | 9.9 | 6.2 | 570 | 1 | 0 | 0 | 296.0 |
| 6 | Kooiker | Male, intact | 8.9 | 11.8 | 374 | 1 | 1 | 0 | 3794.1 |
| 7 | Terrier | Female, sprayed | 10.3 | 4.4 | 124 | 1 | 0 | 0 | 563.4 |

| | | | | | | | | | |
|---|---|---|---|---|---|---|---|---|---|
| 8 | Lagotto Romagnolo | Female, sprayed | 14.2 | 13.0 | 326 | 1 | 0 | 0 | 775.1 |
| 9 | Dachshund | Male, intact | 14.1 | 8.2 | 563 | 0 | 1 | 0 | 1272.9 |
| 10 | Australian Shepherd | Male, castrated | 7.2 | 26.5 | 528 | 0 | 0 | 1 | 2422.0 |
| 11 | French Bulldog | Female, intact | 8.6 | 7.3 | 301 | 1 | 1 | 0 | 2059.0 |
| 12 | Mixed | Male, castrated | 12.8 | 8.1 | 451 | 0 | 1 | 0 | 1346.0 |
| 13 | Yorkshire Terrier | Male, castrated | 10.1 | 5.1 | 414 | 0 | 0 | 0 | 16279.0 |
| 14 | Dachshund | Female, sprayed | 10.2 | 5.2 | 374 | 0 | 1 | 0 | 7642.0 |
| 15 | Mixed | Female, sprayed | 8.2 | 7.4 | 144 | 1 | 1 | 0 | 4247.0 |
| 16 | Jack Russell Terrier | Female, sprayed | 14.3 | 8.2 | 193 | 1 | 0 | 0 | 264.0 |
| 17 | American Staffordshire Terrier | Female, sprayed | 7.9 | 24.0 | 269 | 1 | 1 | 0 | 270.0 |
| 18 | Mixed | Female, sprayed | 9.5 | 23.0 | 278 | 0 | 1 | 0 | 634.0 |
| 19 | Pyrenees Mountain Dog | Female, intact | 11.6 | 72.0 | 245 | 0 | 0 | 0 | 204.0 |
| 20 | Beagle | Female, sprayed | 10.5 | 11.8 | 190 | 1 | 1 | 1 | 236.0 |

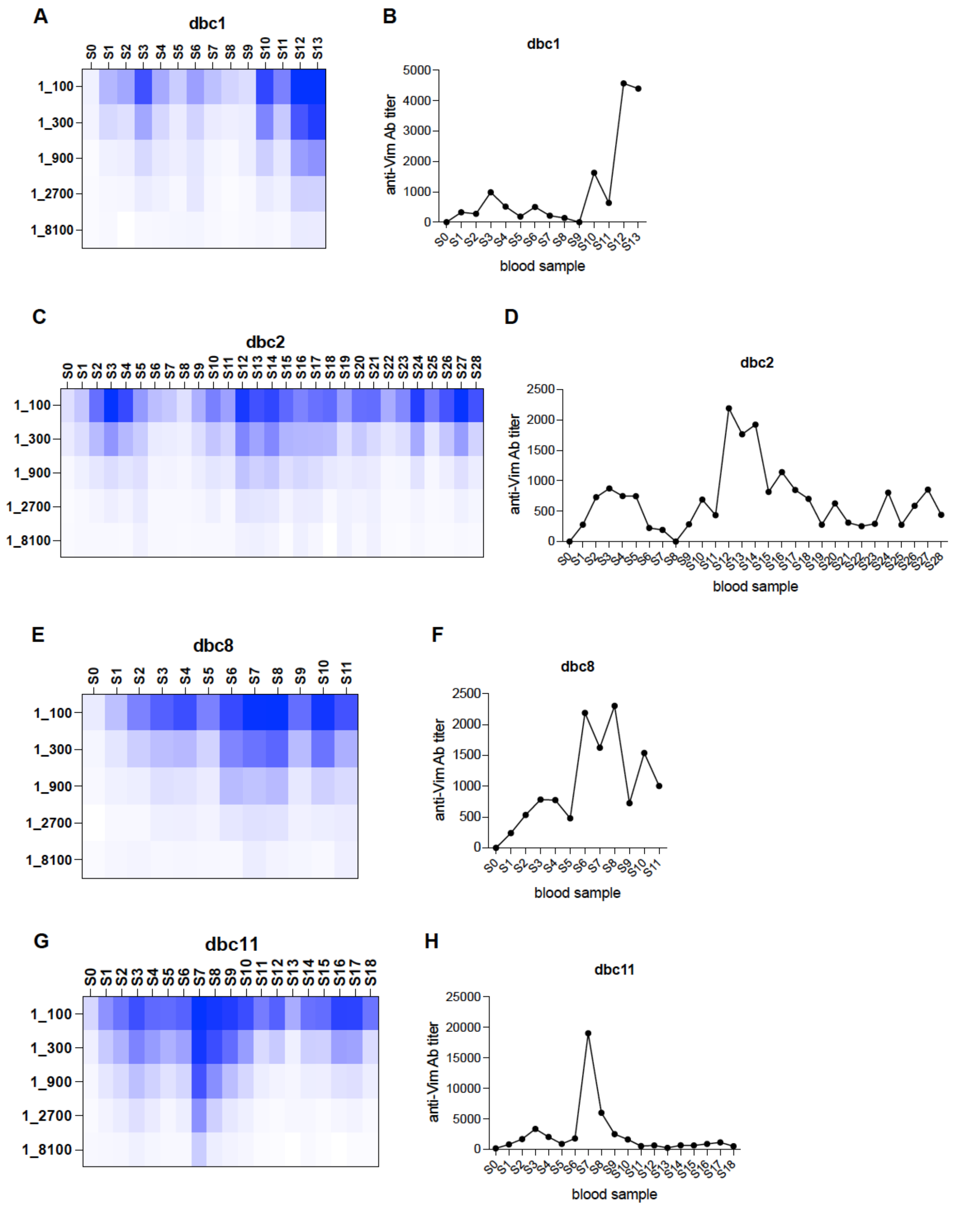


**Figure S2.** Anti-Vim antibody response over time. Heat maps of anti-Vim antibodies measured in canine blood samples (S0-SX) for dog dbc1 (A), dbc2 (C), dbc8 (E) and dbc11 (G). Serum dilutions 1:100; 1:300; 1:900; 1:2700 and 1:8100. Anti-Vim antibody titers measured over time in canine blood samples (S0-SX) for dog dbc1 (B), dbc2 (D), dbc8 (F) and dbc11 (H).

**A**

| | Human | Dog | Mouse |
|---|---|---|---|
| **Human** | | **98.1** | **97.1** |
| **Dog** | ***9*** | | **97.6** |
| **Mouse** | ***12*** | ***11*** | |

**B**

```
mm_Vim      MSTRSVSSSSYRRMFGGSGTSSRPSSNRSYVTTSTRTYSLGSALRPSTSRSLYSSSPGGA    60
hs_Vim      MSTRSVSSSSYRRMFGGPGTASRPSSSRSYVTTSTRTYSLGSALRPSTSRSLYASSPGGV    60
cl_Vim      MSTRSVSSSSYRRMFGGPGTGSRPSSTRSYVTTSTRTYSLGSALRPSTSRSLYASSPGGA    60
            ****************** **.*****.************************:******.

mm_Vim      YVTRSSAVRLRSSVPGVRLLQDSVDFSLADAINTEFKNTRTNEKVELQELNDRFANYIDK    120
hs_Vim      YATRSSAVRLRSSVPGVRLLQDSVDFSLADAINTEFKNTRTNEKVELQELNDRFANYIDK    120
cl_Vim      YATRSSAVRLRSSVPGVRLLQDSVDFSLADAINTEFKNTRTNEKVELQELNDRFANYIDK    120
            *.**********************************************************

mm_Vim      VRFLEQQNKILLAELEQLKGQGKSRLGDLYEEEMRELRRQVDQLTNDKARVEVERDNLAE    180
hs_Vim      VRFLEQQNKILLAELEQLKGQGKSRLGDLYEEEMRELRRQVDQLTNDKARVEVERDNLAE    180
cl_Vim      VRFLEQQNKILLAELEQLKGQGKSRLGDLYEEEMRELRRQVDQLTNDKARVEVERDNLAE    180
            ************************************************************

mm_Vim      DIMRLREKLQEEMLQREEAESTLQSFRQDVDNASLARLDLERKVESLQEEIAFLKKLHDE    240
hs_Vim      DIMRLREKLQEEMLQREEAENTLQSFRQDVDNASLARLDLERKVESLQEEIAFLKKLHEE    240
cl_Vim      DIMRLREKLQEEMLQREEAESTLQSFRQDVDNASLARLDLERKVESLQEEIAFLKKLHDE    240
            ********************.*************************************:*

mm_Vim      EIQELQAQIQEQHVQIDVDVSKPDLTAALRDVRQQYESVAAKNLQEAEEWYKSKFADLSE    300
hs_Vim      EIQELQAQIQEQHVQIDVDVSKPDLTAALRDVRQQYESVAAKNLQEAEEWYKSKFADLSE    300
cl_Vim      EIQELQAQIQDQHVQIDMDVSKPDLTAALRDVRQQYESVAAKNLQEAEEWYKSKFADLSE    300
            **********:******:******************************************

mm_Vim      AANRNNDALRQAKQESNEYRRQVQSLTCEVDALKGTNESLERQMREMEENFALEAANYQD    360
hs_Vim      AANRNNDALRQAKQESTEYRRQVQSLTCEVDALKGTNESLERQMREMEENFAVEAANYQD    360
cl_Vim      AANRNNDALRQAKQESNEYRRQVQSLTCEVDALKGTNESLERQMREMEENFAVEAANYQD    360
            ****************.***********************************:*******

mm_Vim      TIGRLQDEIQNMKEEMARHLREYQDLLNVKMALDIEIATYRKLLEGEESRISLPLPTFSS    420
hs_Vim      TIGRLQDEIQNMKEEMARHLREYQDLLNVKMALDIEIATYRKLLEGEESRISLPLPNFSS    420
cl_Vim      TIGRLQDEIQNMKEEMARHLREYQDLLNVKMALDIEIATYRKLLEGEESRIALPLPNFSS    420
            ***************************************************:****.***

mm_Vim      LNLRETNLESLPLVDTHSKRTLLIKTVETRDGQVINETSQHHDDLE        466
hs_Vim      LNLRETNLDSLPLVDTHSKRTLLIKTVETRDGQVINETSQHHDDLE        466
cl_Vim      LNLRETNLDSLPLVDTHSKRTLLIKTVETRDGQVINETSQHHDDLE        466
            ********:*************************************
```

**Figure S3.** Sequence alignment vimentin. (A) Percentage sequence identity (top right) and number of mismatches (bottom left; italicized) between dog, human and mouse vimentin. (B) Multiple sequence alignment of dog, human and mouse vimentin. Amino acids that differ between species are highlighted.

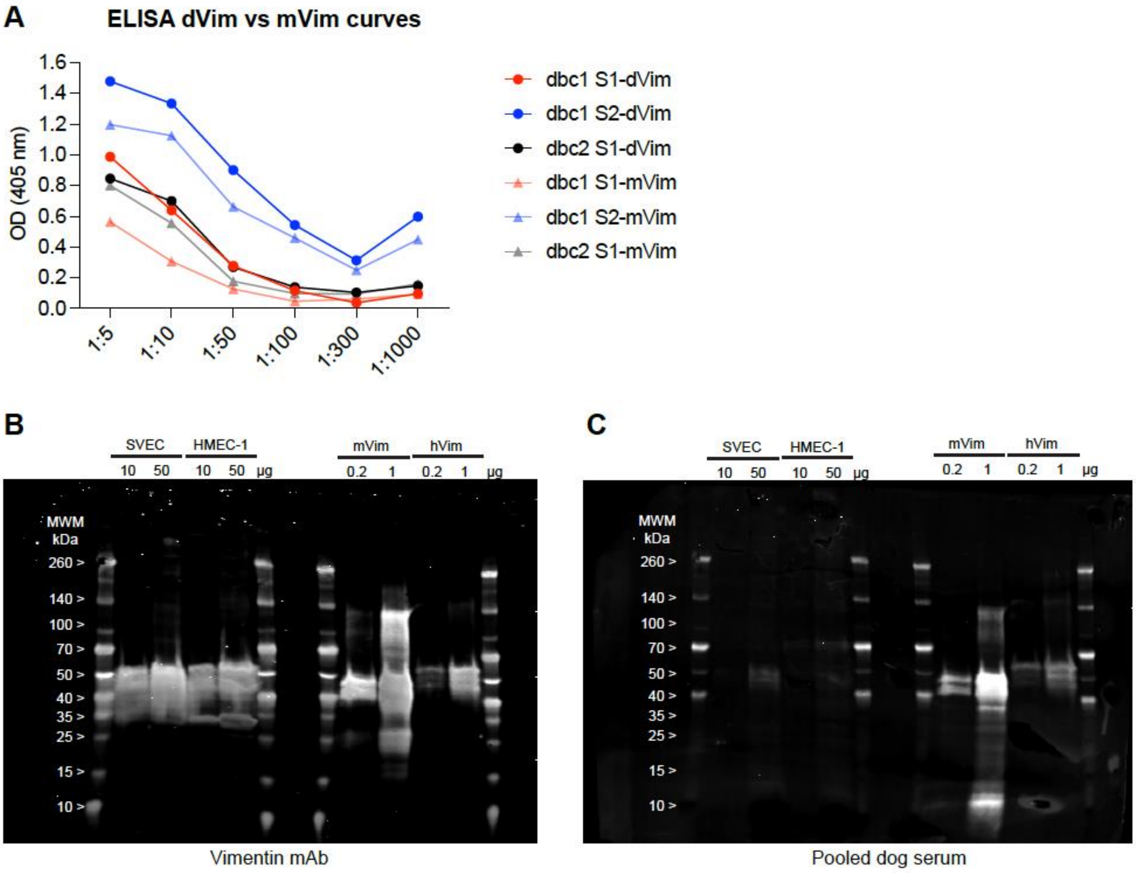


**Figure S4.** Reactivity of canine sera with murine and human vimentin. (A) Comparison of reactivity of the serum of CVx1 vaccinated dogs (dbc1 and dbc2) blood sample S1, S2 with dVim (dog vimentin) or mVim (mouse vimentin). Serum dilutions 1:5; 1:10; 1:50; 1:100 and 1:1000. As expected sera react with both dog and mouse vimentin. (B) Western blot analysis of mouse and human endothelial cell lysates and recombinant vimentin protein. Blots were probed with mouse monoclonal anti-vimentin antibody. (C) Western blot analysis of mouse and human endothelial cell lysates and recombinant vimentin protein. Blots were probed with pooled dog serum. Rather faint yet specific immunoreactivity of the dog serum with cellular proteins at the expected molecular weight of ~55kDa is observed (left part of the blot). The characteristic laddering pattern of vimentin in cell lysates resulting from endogenous protease activity is consistent with previous observations (left parts of blots). Both aggregation (mVim) and laddering (mVim and hVim) can be observed (right parts of blots). At higher loading concentrations of mVim, dog serum also seems reactive with co-purified proteins as the procedure for production of dVim and mVim is identical.

## References

[1] van Beijnum JR, Huijbers EJM, van Loon K, Blanas A, Akbari P, Roos A, et al. Extracellular vimentin mimics VEGF and is a target for anti-angiogenic immunotherapy. Nat Commun 2022;13. https://doi.org/10.1038/s41467-022-30063-7.

[2] Parker R, Deville S, Dupuis L, Bertrand F, Aucouturier J. Adjuvant formulation for veterinary vaccines: Montanide™ Gel safety profile. Procedia Vaccinol 2009;1:140–7. https://doi.org/10.1016/j.provac.2009.07.026.
